# Pulse-Atomic Force Lithography: A Powerful Nanofabrication Technique to Fabricate Constant and Varying-Depth Nanostructures

**DOI:** 10.3390/nano12060991

**Published:** 2022-03-17

**Authors:** Paolo Pellegrino, Alessandro Paolo Bramanti, Isabella Farella, Mariafrancesca Cascione, Valeria De Matteis, Antonio Della Torre, Fabio Quaranta, Rosaria Rinaldi

**Affiliations:** 1 Department of Mathematics and Physics “Ennio De Giorgi”, University of Salento, Via Monteroni, 73100 Lecce, Italy; mariafrancesca.cascione@unisalento.it (M.C.); valeria.dematteis@unisalento.it (V.D.M.); ross.rinaldi@unisalento.it (R.R.); 2 Lecce Labs, STMicroelectronics S.r.l., System Research and Applications (SRA) Silicon Biotech, Via Monteroni, 73100 Lecce, Italy; 3 Institute for Microelectronics and Microsystems (IMM), CNR, Via Monteroni, 73100 Lecce, Italy; isabella.farella@cnr.it (I.F.); antonio.dellatorre@cnr.it (A.D.T.); fabio.quaranta@cnr.it (F.Q.)

**Keywords:** atomic force-nanolithography, tip-based nanolithography, atomic force microscopy, nanofabrication, varying-depth nanostructures

## Abstract

The widespread use of nanotechnology in different application fields, resulting in the integration of nanostructures in a plethora of devices, has addressed the research toward novel and easy-to-setup nanofabrication techniques to realize nanostructures with high spatial resolution and reproducibility. Owing to countless applications in molecular electronics, data storage, nanoelectromechanical, and systems for the Internet of Things, in recent decades, the scientific community has focused on developing methods suitable for nanopattern polymers. To this purpose, Atomic Force Microscopy-based nanolithographic techniques are effective methods that are relatively less complex and inexpensive than equally resolute and accurate techniques, such as Electron Beam lithography and Focused Ion Beam lithography. In this work, we propose an evolution of nanoindentation, named Pulse-Atomic Force Microscopy, to obtain continuous structures with a controlled depth profile, either constant or variable, on a polymer layer. Due to the modulation of the characteristics of voltage pulses fed to the AFM piezo-scanner and distance between nanoindentations, it was possible to indent sample surface with high spatial control and fabricate highly resolved 2.5D nanogrooves. That is the real strength of the proposed technique, as no other technique can achieve similar results in tailor-made graded nanogrooves without the need for additional manufacturing steps.

## 1. Introduction

The development of nanostructures has gained increasing attention in the scientific community, motivated by the variety of applications in a number of fields, including industrial and medical applications. To be usable in real-world applications, however, nanostructures need to be highly reproducible and accurate, while requiring inexpensive fabrication protocols.

Today, Tip-Based Nanofabrication (TBN) is a fast-growing alternative in the category of top-down nanofabrication, thanks to its flexibility to sculpt, in a single step, either hard material substrate, polymers, or biological materials [1,2]. TBN uses a nanometric tip brought into contact or proximity to the substrate surface to be modified, exploiting a feedback loop apparatus, which controls the relative position between the tip and the surface. The physical modification of the surface can be induced using thermal, mechanical, or electrical fields as described in depth in the review by Hu et al. [3].

Among these, mechanical TBN (m-TBN) is one of the most promising techniques in the realization of two- and three-dimensional nanofeatures. The m-TBN can be performed using a Scanning Tunnelling Microscope (STM), an Atomic Force Microscope (AFM), and a nanoindenter; however, the m-TBN based on Atomic Force Microscopy (AFM) is certainly the most interesting, due to its high versatility. In fact, it enables patterning on different substrates (hard, soft, and biological substrates) with high resolution and in a single step [4], thus, overcoming the limitations imposed by the chemical and thermal effects of the conventional mechanical removal process.

To date, the AFM tip can sculpt the surface through three different approaches: Nanoindentation, Static Plowing Lithography (SPL), and Dynamic Plowing Lithography (DPL) descending from indentation [4,5], contact [6,7], and tapping scanning modes [8,9,10,11], respectively.

Operating in contact, nanoindentation is commonly used to investigate the superficial mechanical properties of materials (e.g., stiffness and adhesion) [4,12]; nevertheless, by applying a large indentation force, it is possible to deform the surface permanently, thereby, patterning arrays of nanodots and pits [13,14,15]. The sizes and shapes of the features depend on the AFM tip geometric profile, the normal force applied, and the rheological characteristics of the material [1,16].

Conversely, in SPL (or nanoscratching), the tip applies a high, constant contact force to the sample surface to scratch lines and geometrical shapes. It also undergoes high friction, that causes relevant cantilever torsion [11], resulting in non-uniform depth profiles, irregular edges of the nanostructures [8], and rapid tip wear.

To overcome these drawbacks in soft materials, working in semi-contact mode is more convenient. In this mode, named Dynamic Plowing Lithography (DPL), the tip punctually shapes the surface by vibrating at a frequency close to resonance, maintained by a closed-loop system [8]. In addition, DPL prevents the cantilever torsion enabling to pattern the substrate with a more uniform depth profile. Unfortunately, this technique requires a long working time and enables only few nm sculpting depths [15].

In order to improve the quality and the shape of two- and three-dimensional nanostructures [17,18], the m-TBN has been coupled with additional support methods, such as ultrasonic vibrating [19,20,21], high tip temperature [22,23,24], rotation of the tip during the milling [25,26] and bias voltage [7]. However, these hybrid processes require more elaborated equipment and are slower and more complex than the conventional m-TBN techniques; in addition, they can target only specific materials [27].

In this work, we propose an alternative m-TBN method, called Pulse Atomic Force Lithography (P-AFL), to pattern nanochannels with nanometric precision and accuracy on a polymeric layer. Through our method, it is possible to quickly obtain 2.5D nanogrooves with desired depth (constant or variable), length, and slope in a single pass and without additional energy sources.

P-AFL can operate in two different modes: Constant Pulse-Atomic Force Lithography (CP-AFL) and Gradient Pulse-Atomic Force Lithography (GP-AFL), which enables the fabrication of nanogrooves with constant or gradient depth profiles, respectively. In the latter case, the grooves change their profile smoothly and continuously. That represents a significant step forward in the nanofabrication field: to date, only the Grayscale Electron Beam Lithography (GEBL) [28] and ultrasonic-vibration-assisted AFM nanomachining [29,30] permit to obtain three-dimensional nanostructures with a varying-depth profile. However, their profiles are stepped unless subsequent smoothing is applied, such as by thermal reflow. Moreover, in some cases, these techniques do not guarantee high reproducibility.

Finally, we demonstrate how combining the P-AFL technique on a polymer with a plasma etching process, the nanochannels were transferred onto an underlying harder material with high fidelity.

## 2. Materials and Methods

### 2.1. Substrate Fabrication

As the substrate, a 4 inch standard (100) silicon wafer with an 0.5 µm thermally grown silicon dioxide layer was used. The wafer was first cleaned by sequential sonication bath in acetone and 2-propanol for 15 min each and then rinsed under flowing deionized water and dried under nitrogen gas stream for 30 s. Both acetone (99.5%) and 2-propanol (99.5%) were purchased from Sigma-Aldrich and used without further dilution. Then, a thin silicon nitride film (SiN) 200 nm-thick was deposited by Plasma-Enhanced Chemical Vapour Deposition (PECVD) in a Surface Technology System (STS) reactor operating at a frequency of 13.56 MHz.

Silane and ammonia were the reactant gases, with nitrogen as the diluent. The SiH_4_, NH_3_, and N_2_ flows were 40, 55, and 1960 sccm, respectively; during the process, the temperature, radio frequency power, and the pressure parameters were fixed to 300 °C, 30 W, and 900 mTorr, respectively. Successively, a thin layer of Polymethyl methacrylate (PMMA) (950 kDa, in solvent anisole), ordered from MicroChem, was spun at 4000 rpm for 30 s on SiN by means of a semiautomatic spinner DELTA 80T (SUSS MicroTec Corp, Germany) and baked on a hotplate at 180 °C for 90 s for solvent dry-out. A test pattern was suitably prepared to measure the thickness of the PMMA; its value, about 60 nm, was measured with an Alpha-Step P6 profilometer (KLA-Tencor Corporation, Milpitas, CA, USA).

### 2.2. Instrumentation for Nanolithography, AFM, and SEM Characterization

AFM lithographies and surface sample characterization were conducted at ambient conditions (room temperature of about 25 °C and relative humidity around 50%) by means of a commercial AFM NTEGRA by NT-MDT Co. (NT-MDT Spectrum Instruments, Moscow, Russia), equipped with spectroscopy and nanolithography moduli. Doped, diamond-coated conductive probes (DCP20, NT-MDT Spectrum Instruments, Moscow, Russia) were used for the experiment in contact mode: both to perform all nanolithography test and to estimate the stiffness of the pile-ups. DCP20 probes are V-shaped cantilevers, with conical tips 10–15 µm high at the apex. The cone angle is less than 22°, and the typical curvature radius is approximately 100 nm. The nominal spring constant (*k*) is 65 N/m, and the resonant frequency is 420 kHz. Before the experiments, the DPC20 cantilevers were calibrated using the thermal noise method [31,32,33]. The force constant was determined to be (62.95 ± 0.9) N/m.

Moreover, NSG01 (NT-MDT Spectrum Instruments, Moscow, Russia) AFM tips were used to both characterize in semi-contact mode the substrates before and after the nanolithography process. NSG01 probes were chosen for the high-resolution characterization of pristine and patterned substrates. The NSG01 probe is a rectangular-shaped cantilever with a much smaller tip curvature radius (~6 nm) and a nominal spring constant of 5 N/m.

The topographic AFM images, the Force-spectroscopy, and the indentation curves were analysed using the Image Analysis P9 (NT-MDT Co.) software. The NOVA_PX software (NT-MDT Co.) was used, instead, for the nanolithography experiments, the DCP20 force constant estimation, and all the morphological characterizations. Both IA-P9 and NOVA_PX were obtained from NT-MDT Spectrum Instruments, Moscow, Russia.

In addition, Scanning Electron Microscopy (SEM) imaging by SEM JEOL model JSM-6500F (JEOL company, Tokyo, Japan) was performed on the nanogrooves after their transfer by ICP etching on silicon nitride substrate.

### 2.3. Nanolithography Methods

Based on Nanoindentation, CP-AFL and GP-AFL nanofabrication methods have been developed to engrave nanogrooves with constant depth and varying depth profile on PMMA, respectively. The two following subsections provide details on the two methods.

#### 2.3.1. Constant Pulse-Atomic Force Lithography

In CP-AFL, the AFM tip is brought into contact with the sample surface, and a voltage pulse of constant amplitude is fed to the piezo-scanner. The pulse makes the scanner extrude along the *z*-direction, causing the tip to penetrate the sample surface. Triangular voltage pulses (symmetric) of duration τ = 100 ms were chosen. During the first τ/2 ms, the piezo-scanner lifts causing the tip to penetrate the sample until the deflection of the cantilever reaches a defined value of setpoint.

**Figure 1:**
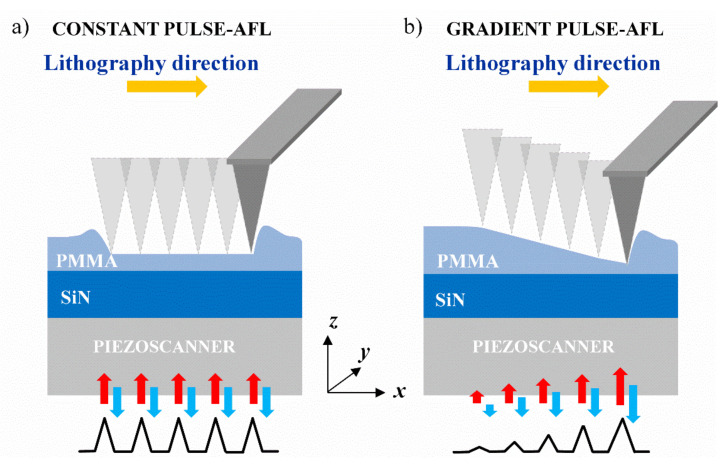
Schematic diagram of (**a**) Constant Pulse- and (**b**) Gradient Pulse- Atomic Force Lithography techniques performed on a thin PMMA layer. The yellow arrow indicates the nanolithography direction, while the red and blue arrows the movement of the piezo-scanner in the z direction, induced by the voltage pulses.

The tip plastically deforms the surface leaving a nanohole of the desired depth. In the remaining τ/2 ms, the scanner returns to its initial position. Since the amplitude of the pulse applied to the piezo-scanner is strictly related to the cantilever deflection, it is possible to control the depth of the nanoholes by properly choosing the setpoint values. A sketch representation of the CP-AFL method is reported in Figure 1a. In our experiments, ten different setpoint values, ranging from 1 to 10 nA in step of 1 nA, were set in the lithography tests in order to find the best values for the desired tailoring of the grooves morphology.

After the pulse, the sample moves across the *xy* plane to the next position to be indented, according to the line template previously designed by the machine software. In order to fabricate a continuous line, the distance between two neighbouring indentation points was fixed as equal to 10 nm, corresponding to the minimum step value allowed by the piezo-stage of our instrument. During CP-AFL, the amplitude, frequency, and shape of the voltage pulses were steadily monitored with an oscilloscope. The movements in the *xy* plane were strictly controlled by the *xy* feedback loop of the AFM piezoelectric scanner.

#### 2.3.2. Gradient Pulse-Atomic Force Lithography

Unlike CP-AFL, in GP-AFL a train of voltage pulses with increasing amplitude was applied to the piezo-scanner, and nanogrooves with a graded depth profile were obtained (Figure 1b). By means of the NOVA_PX software, it was possible to appropriately set the value of starting and final setpoints, indentation step, and the length of the nanogroove.

For all the nanogroove lengths, the range of setpoints was chosen to linearly increase from 0 to 10 nA to obtain sloped profiles. To ensure that neighbouring indentations overlapped effectively, the same step of 10 nm was chosen. As for CP-AFL experiments, the train of the voltage pulses was constantly monitored by an oscilloscope, and the *xy* feedback loop of the piezoelectric scanner ensured the accurate movement in the *xy* plane.

### 2.4. Substrates Characterization by Atomic Force Microscopy (AFM)

2D and 3D AFM images of PMMA and SiN layers were acquired in the SensHeight channel, which is more sensitive to the surface landscape than the Height channel, with a resolution of 1024 × 1204 points for substrates, while 768 × 768 points for nanostructures, by using NSG01 tips. The setpoint and gain parameters used in the AFM topography acquisition were set to 5.1 and 0.25 nA, respectively. The raw topographic AFM images were firstly deconvoluted, and then a second order plane fit was applied to each topographical image in order to remove every artifact, like bow and three-dimensional effects, by IA-P9 software. The 2D and 3D root mean square roughness (R_q_) were estimated on the AFM topographical images with the ISO 4287 method [34].

### 2.5. PMMA Stiffness Estimation

To quantify the stiffness of the PMMA substrates and border pile-ups after CP-AFL and GP-AFL processes, force indentation curves were acquired with DCP20 tips. The force-distance curves were fitted with the Sneddon model by means of Image Analysis P9 (IA-P9), and Young’s modulus (*E*) was obtained as the best fit parameter. The *E* was calculated as average over 10 indentations curves and expressed as the mean value ± SD.

### 2.6. Transfer of Nanostructures Via ICP Etching

At first, the nanogrooves with constant- and gradient-depth profiles were fabricated on PMMA by CP- and GP-AFL, according to previously reported experimental conditions. Successively, using the PMMA as a mask, the nanogrooves were transferred on the underlying SiN layer via a dry etching process. The nanopatterned samples were etched in a Surface Technology Systems (STS) multiplex ICP system using SF_6_/O_2_ gas mixtures (100/10 sccm) for 120 s at an ICP electrode power of 10 W, with no coil excitation, thus, in RIE configuration, as we sought for very mild etching.

### 2.7. Statistical Analysis

All the results were expressed as mean values and associated standard deviation. The difference among data was analysed through ANOVA multiple comparisons. The differences were statistically significant when * *p* < 0.05, ** *p* < 0.01, and *** *p* < 0.001).

## 3. Results

**Figure 2:**
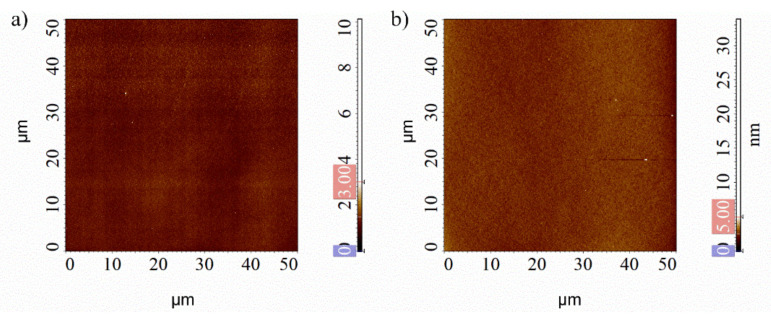
High resolution SensHeight AFM images of silicon nitride (**a**) and PMMA (**b**) surfaces.

Before the PMMA samples surface morphology characterization, the topography of the silicon nitride surface substrate was investigated by AFM equipped with NSG01 tip in semi-contact mode: the surface appeared quite smooth (Figure 2a). After PMMA spin-coating deposition, no appreciable modification of the sample roughness was observed (Figure 2b). The root-mean-square surface roughness (R_q_) value, calculated over fifteen areas (5 × 5) µm^2^ wide, remained comparable, changing from (0.26 ± 0.09) nm to (0.20 ± 0.02) nm after PMMA deposition.

Prior to fabricating the nanochannels by the CP-AFL technique, it was necessary to estimate the normal force acting on the AFM tip by the force-spectroscopy; this step aimed to identify the proper setpoint value to pattern nanochannel with desired depth. For this purpose, force-spectroscopy analysis was performed in contact mode: by analysing the force-distance curves that correlated the cantilever deflection with the piezo-scanner extension, the normal force exerted on the tip was calculated for setpoint values, ranging from 1 to 10 nA, in steps of 1 nA. For each setpoint, the corresponding normal force was estimated by averaging over 20 force-distance curves, acquired by a DCP20 tip on a silicon nitride substrate. The mean values and corresponding standard deviations of the forces are reported in Table 1.

In correspondence to each setpoint value, a nanochannel was sculpted in contact mode along an orthogonal direction to the cantilever main axis by CP-AFL by means of the DCP20 tip.

**Figure 3:**
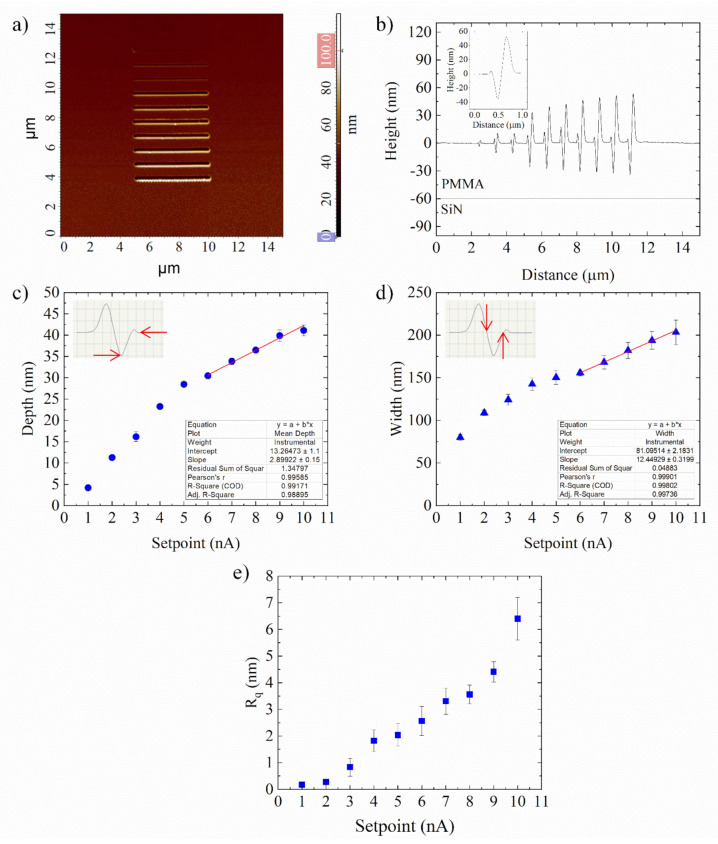
(**a**) SensHeight AFM images of 5 µm long nanogrooves patterned on a PMMA surface and the corresponding cross section profiles (**b**); in the inset, the V-shaped profile of groove corresponding to 10 nA setpoint (17.94 µN force). Depths (**c**) and widths (**d**) of nanogrooves on PMMA for each setpoint. The definition of measured depths and widths are schematically shown in the respective insets. Red line for depths (**c**) and widths (**d**) is best linear fit for higher setpoint value (6 nA ÷ 10 nA). (**e**) Roughness (R_q_) mean values, obtained by averaging 10 different point in the channels manufactured setting different setpoints. The data reported in (**c**–**e**) were statistically significant for *p* < 0.01.

The obtained array of nanochannels was imaged by NSG01 probe in semicontact mode, in order to prevent further mechanical deformations of the pattern. The 2D AFM images of the nanogrooves array in the PMMA layer and the corresponding cross-section profile are reported in Figure 3a,b, respectively. Nanogrooves patterned on PMMA were contoured along their length with high edges. These bulges became more evident in deeper grooves (higher setpoints), whereas they almost vanished in shallow lines, as is evident from cross-section profiles (Figure 3b).

The cross-section channel profiles were V-shaped, as shown in the inset of Figure 3b, which refers to the line indented with the highest setpoint (10 nA, force equal to 17.94 µN). For each nanochannel on PMMA, corresponding to a fixed setpoint, the experimental depth and width were estimated by averaging their values at twenty-five different locations randomly selected on the AFM images (Figure 3c,d).

As it can be seen, the increase of the normal force from 2.14 µN (setpoint 1 nA) to 17.94 µN (setpoint 10 nA) caused an increase in the depth of the nanogrooves from 4.19 ± 0.5 nm to 41.1 ± 1.2 nm. In the range of high forces 10.91 ÷ 17.94 µN, an almost linear increase of groove depth (Figure 3c), as well as width (Figure 3d) was observed, though it is less steep than in the initial force range. Jiang et al. reported a similar trend in nanochannels scratched directly by SPL on a silicon substrate.

The authors described this experimental finding by assuming a decrease of the effective normal force acting on the tip during tip scratching, as the contact area of the conical tip with the sample becomes larger [35]. Consequently, the increase of the contact area for the larger setpoint values could cause an increase in the adhesion and friction forces on the tip, resulting in a less steep linear behaviour. Regarding the width of channels (Figure 3d), it increased with the setpoint value from 79.9 ± 3.3 nm to 213.3 ± 14.3 nm, since increasing setpoints (greater forces) correspond to deeper AFM tip penetration and wider channels. In addition, the roughness of the PMMA nanochannels was estimated by measuring the 2D R_q_ parameter. Independently of the depth of the channels, the roughness was very low, ranging from 0.17 ± 0.07 nm to 6.4 ± 0.8 nm (Figure 3e).

**Figure 4:**
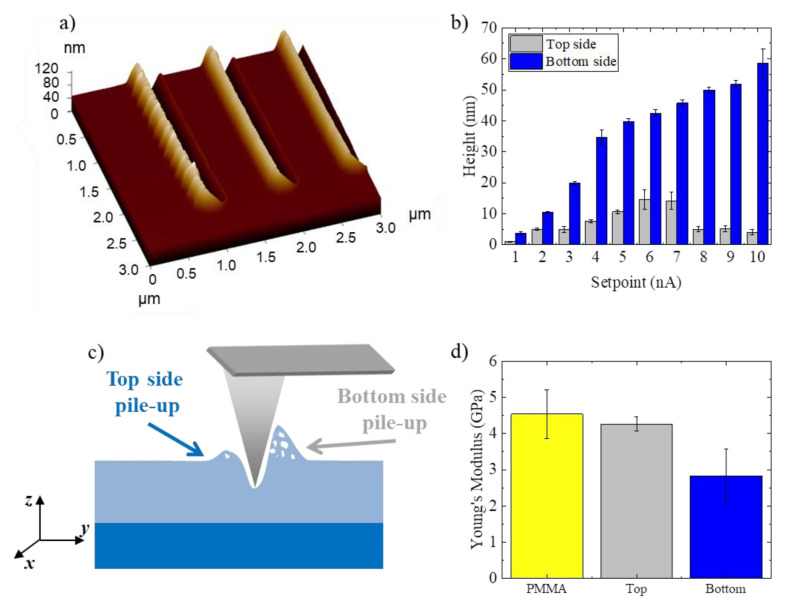
(**a**) 3D AFM topographic image of the deepest nanogrooves. (**b**) Heights of the PMMA pile-ups after nanolithography. (**c**) Schematic representation of the pile-ups formation with cracks. (**d**) Young’s modulus values of the raw PMMA, bottom and top pile-ups. The reported mean values ± SD were calculated for 10 different measures. The data reported were statistically significant for *p* < 0.05.

As aforementioned and clearly seen in Figure 3a and Figure 4a, during nanolithography pile-ups accumulate around the nanochannels. The tip induces a plastic deformation of the PMMA causing the displacement of material from the nanogroove and, thus, its accumulation at the contours of nanochannels (Figure 4a). With respect to nanolithography direction (i.e., along the channel), the hillock of material accumulated on the bottom side (bottom pile-up) is always higher than the corresponding upper one for all the nanochannels.

That height difference was due to the movement of the tip, which dug the line along a direction normal to the cantilever axis. Thus, the tip was asymmetrically bent with respect to the groove, digging more deeply close to one border than to the other (Figure 4a,c).

Moreover, the pile-up height was proportional to the setpoint (Figure 4b). It can also be observed that the height of the bulges at the bottom side of the grooves was greater compared to the nanogroove depth. This led us to suppose that the bulges material was not compact, presenting fractures or cracks inside (Figure 4c). This was likely due to the rapid local modification of the surface by the indenter, whose tip pushed the polymer removed from the nanochannel along its edges, where it accumulated. Then, pile-ups raised up fast at the grooves borders with cracks inside.

In order to confirm this hypothesis, Young’s modulus of the pile-ups and raw PMMA was estimated by acquiring indentation curves with DCP20 tips. The Young’s modulus (E) of the raw PMMA surface resulted equal to 4.54 ± 0.68 GPa, while the E of the pile-ups at the top and bottom side were 4.26 ± 0.2 GPa and 2.83 ± 0.73 GPa, respectively (Figure 4d). The similar E values found in raw PMMA and top-side pile-ups (shorter bulges) led us to suppose that the latter did not have cracks; while the bottom pile-ups (higher bulges), being less stiff than raw PMMA, could have fractures. Our hypothesis was corroborated by similar results obtained by Yan and co-workers. In the SPL nanolithography experiment performed on polycarbonate, in detail, they demonstrated how the stiffness of the pile-ups depends on the presence of internal cracks [14].

Up to this point, we optimised the lithography procedure on the PMMA surface to fabricate constant height channels in contact mode. We characterised the geometrical parameters of the obtained channels with reference to the type of pulse applied both to the piezoelectric scanner operating along the *z*-axis (voltage pulse amplitude and pulse width) and to the scanner operating in the *xy* plane (step, i.e., the distance between successive indentations). Then, our efforts focused on the nanoengraving of variable depth nanochannels.

**Figure 5:**
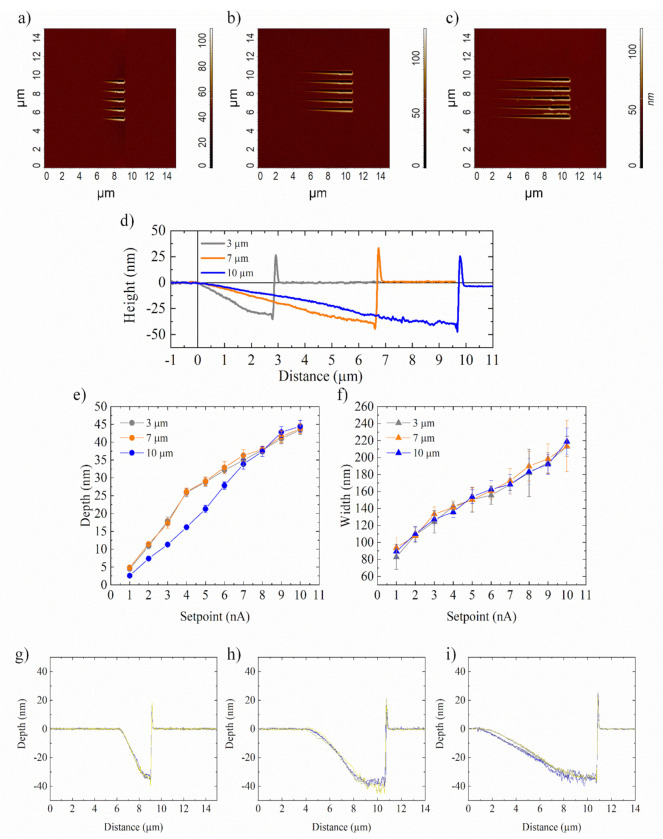
SensHeight AFM topography of nanochannels with a linearly variable depth profile, 3 (**a**), 7 (**b**), and 10 µm (**c**) long. (**d**) Representative cross-section profile of nanochannels 3, 7, and 10 µm long. (**e**) Mean depth and (**f**) width values in correspondence to different setpoint; each mean value was calculated for 20 points. (**g**–**i**) The superimposition of five different cross-section curves for nanochannels 3 (**g**), 7 (**h**), and 10 µm (**i**) long. The data reported in (**e**,**f**) were statistically significant for *p* < 0.001.

To this aim, we developed the GP-AFL lithography protocol. In GP-AFL, a continuously varying voltage pulses train was applied to the piezo-scanner. In contrast to the CP-AFL, in GP-AFL the amplitude of the voltage pulse could no longer assume a constant value; rather, it had to continuously vary between a minimum and a maximum setpoint value (in our case, 0 and 10 nA, respectively). In this way, nanochannels with linearly variable depth profiles were achieved (Figure 5a–c).

Our results obtained analysing the cross-section of nanochannels (Figure 5d) confirmed that the overall depth variation, from the sample surface to the maximum depth, was the same in the 3, 7, and 10 µm long nanochannels, being 43.5 ± 1.3, 43.8 ± 1.4, and 44.5 ± 1.7 nm, respectively (Figure 5e), for fixed setpoint = 10 nA. Clearly, the different channel lengths induced a variation in the slope of the depth profile (Figure 5d). In detail, the angles were equal to 0.98° ± 0.05°, 0.58° ± 0.03°, and 0.27° ± 0.02° for the 3, 7, and 10 µm long nanochannels, respectively.

The nanogrooves width changed along the channel in correspondence to the different setpoints (Figure 5f). As in the case of the CP-AFL fabrication method, the nanochannels enlargement in correspondence to higher setpoint value was due to an increase of the penetration rate of AFM tip, which had a conical shape profile.

The lithography experiment was replicated five times, and the profiles obtained were clearly superimposable (Figure 5g–i), thus, indicating that the optimised protocol was highly reproducible. Finally, the morphological characterization was completed quantifying the R_q_ mean value for each channel: from 3 to 10 µm long lines, we measured 0.8 ± 0.4, 1.6 ± 0.5, and 2.9 ± 1.1 nm.

**Figure 6:**
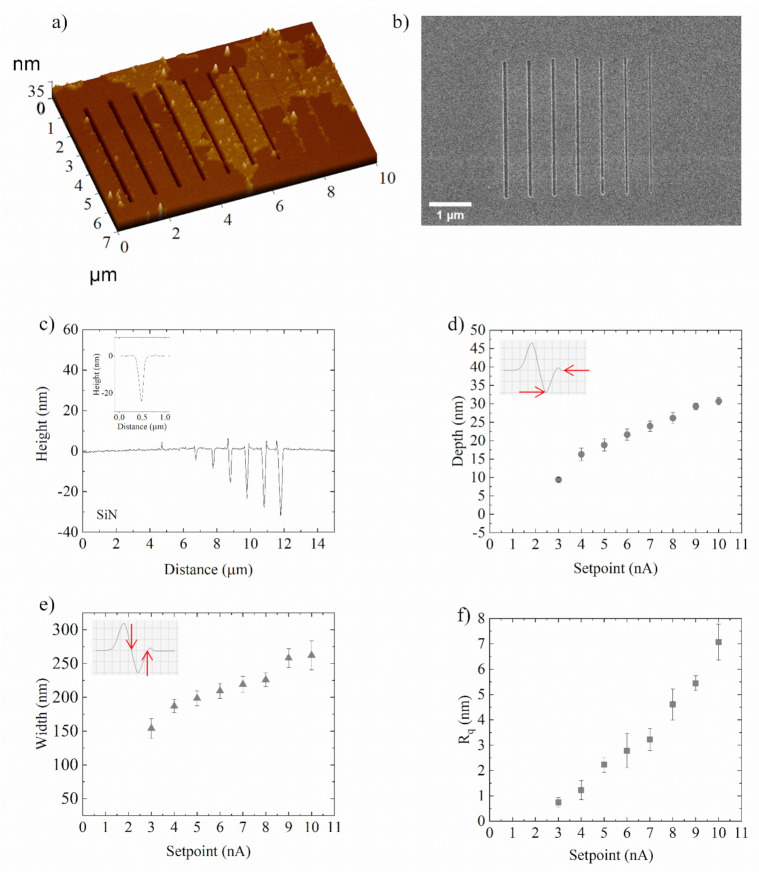
(**a**) Topview SensHeight AFM and (**b**) SEM images of 5 µm long nanogrooves, obtained by means of CP-AFL, transferred from PMMA to SiN via ICP etching, and (**c**) corresponding cross section profiles; the V-shaped profile of deeper nanogrooves was reported in the inset. (**d**) Depths and (**e**) widths of nanogrooves with standard deviations. The definition for measured depths and widths are schematically shown in the respective insets. (**f**) Roughness values. Setpoint indicated as x-axis in (**d**–**f**) graphs referred to channels obtained on PMMA using that setpoint value and then transferred by etching onto the SiN substrate. The scale bar in the SEM image is 1 µm long. Data reported in figure (**d**–**f**) were statistically significant for *p* value < 0.001.

Finally, the transfer of the nanochannels made on the PMMA to the underlying SiN substrate, by means of the ICP etching process, was tested. As previously mentioned, we first optimised the etching procedure for channels with a constant depth profile, and then we checked its validity on those with a linearly variable profile. In order to remove the traces of PMMA after the etching process, the samples were immersed in hot acetone and successively in hot isopropanol for 15 min, respectively. Then, the samples were washed under deionized water and rinsed under N_2_ flow. After that, the nanostructures were characterized by AFM at high resolution (Figure 6a) and SEM (Figure 6b).

The pile-ups were almost completely removed (Figure 6a,b); however, PMMA residues still remained on the SiN substrate. The lines transferred onto the SiN substrate appeared better defined than their PMMA counterparts for the nanochannels achieved with a setpoint value greater than 3 nA. In fact, the shallowest lines on the PMMA, engraved with a setpoint value of 1 and 2 nA, were not accurately reproduced in the SiN layer (Figure 6a–c). This was likely due to the different etch rate of PMMA (~0.47 nm/s) against SiN (~0.38 nm/s) materials.

In addition, the V-shape of the nanochannels was successfully reproduced on SiN (Figure 6c). The depth and width of each channel transferred on the SiN substrate were estimated following the same procedure used in the case of PMMA channels. The different etch rates on PMMA and SiN also justified the overall slight decrease of the channel depth in the SiN substrate, from 9.4 ± 0.6 nm to 30.7 ± 1.1 nm (Figure 6d). On the other hand, the isotropy of the etching process caused an enlargement of channel width in SiN, varying from 153.9 ± 14.6 nm to 262 ± 21.3 nm (Figure 6e) with respect to the maximum width of PMMA nanochannels (about 213 nm, as reported in Figure 3e).

As in the case of the manufactured nanochannels in PMMA, the 2D roughness estimation in terms of R_q_ value was quantified into SiN transferred nanochannels (Figure 6f): the channel surface appeared very flat since the R_q_ ranged from to 0.2 ± 0.1 nm to 7.1 ± 0.7 nm. These results were comparable to those achieved in the PMMA case, suggesting a good fidelity of the etching process in transferring nanochannels from PMMA to SiN substrate.

**Figure 7:**
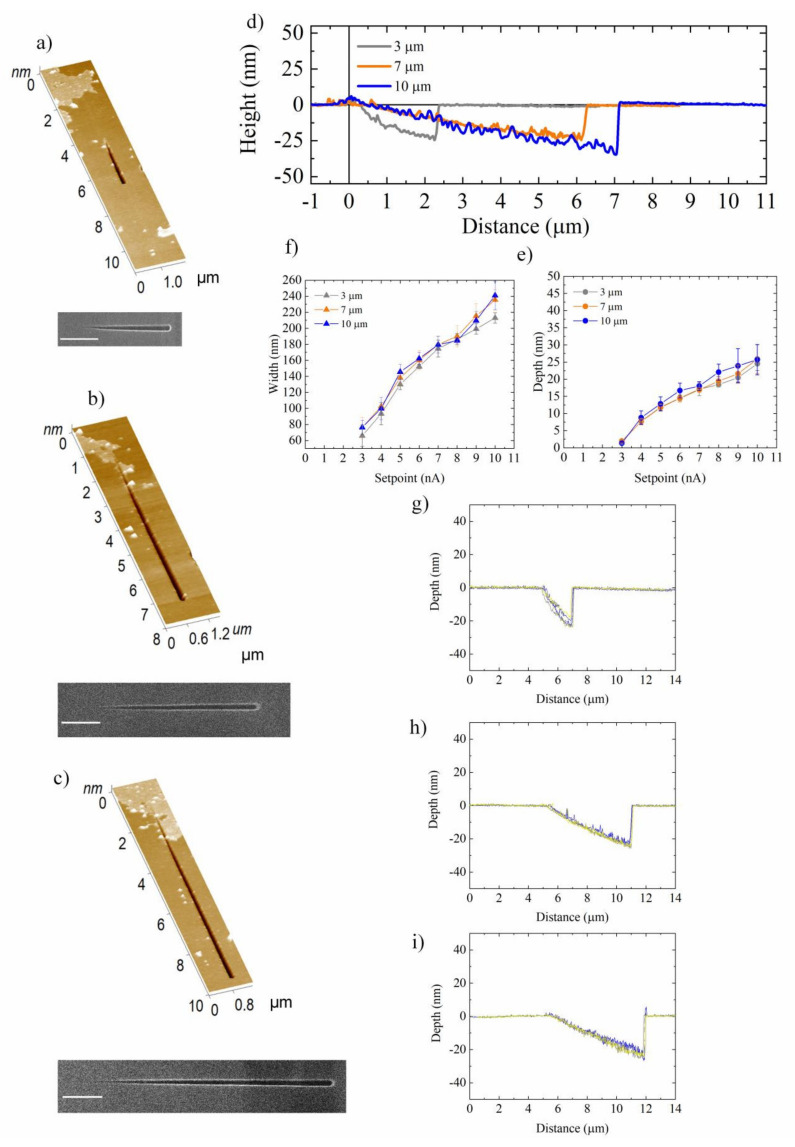
Results of analysis performed on nanochannels with linearly variable depth profile transferred onto the SiN substrate by means ICP etching. SEM and SensHeight AFM 3D-topography of nanochannels with linearly variable depth profile 3 (**a**), 7 (**b**), and 10 µm (**c**) long. The scale bars in the SEM images are 1 µm long. (**d**) Superimposition of representative cross-section profile of nanochannels 3, 7, and 10 µm long (**e**) Depth and (**f**) width values in correspondence to different setpoint; each mean value was calculated on 20 points. (**g**–**i**) show the superimposition of five different cross-section curves in the case of nanochannels 3 (**g**), 7 (**h**), and 10 µm (**i**) long. Data reported in (**e**,**f**) were statistically significant for *p* < 0.01.

The etching procedure optimized to transfer the nanochannel obtained by CP-AFL mode and the following cleaning in acetone and isopropanol were adopted to reproduce the 3, 7, and 10 µm long nanochannels, manufactured by GP-AFL on the SiN substrate (Figure 7a–c). From a preliminary observation of the topographic AFM and SEM acquisitions, it was clear that nanochannels transferred onto the SiN substrate were shorter than their counterparts developed in PMMA.

This result was not unexpected in light of the analogous CP-AFL results at low setpoints (1 and 2 nA). Indeed, the first portion of the varying-depth nanochannels (Figure 7a–c), corresponding to the lowest setpoint values, were not reproduced onto the underlying material, giving rise to shorter nanostructures. However, the length reduction of nanochannels could be compensated by properly optimizing the Gradient Pulse nanolithography parameters.

Moreover, the analysis of cross-sections highlighted the preservation of the continuously variable profiles from PMMA to SiN (Figure 7d). As expected, the overall depth change—equal to the total depth difference between the minimum and maximum setpoints—was about 25 nm for all lengths (Figure 7e). Nevertheless, the angles were smaller than in their PMMA counterparts, being equal to (0.61 ± 0.18)°, (0.26 ± 0.03)°, and (0.12 ± 0.02)° for 3, 7, and 10 µm lines, respectively. In addition, as already observed and justified in previously described CP-AFL experiments, the channels transferred on SiN exhibited a width enhancement (Figure 7f).

Lastly, to completely characterize the nanochannel morphology, the R_q_ mean value was estimated obtaining (1.1 ± 0.3), (1.8 ± 0.6), and (2.7 ± 0.5) nm for nanochannels of 3, 7, and 10 µm, respectively. These results compare well with their counterparts in PMMA.

Based on the results above, our nanolithographic procedures are promising for obtaining continuous structures with a constant or variable depth profile on a thin polymer layer. Although the proposed approach is lower in throughput than conventional nanolithography, several features make P-AFL suitable for widespread usage, namely, the low cost of the instruments used, the ease of the lithographic transfer, the possibility of inspecting and characterizing the structures immediately after patterning by the same instrument used for fabrication, the operability in ambient conditions, and, above all, the capability of patterning 2.5D nanostructures.

In addition, the possibility to transfer the nanochannels onto an underlying harder material, with high fidelity, is a starting point for developing nanostructures integrable in devices where a stack of dielectrics with varying-depth profiles is required, such as for molecular computing [36,37,38], as recently proposed and patented [39]. Due to their accuracy further employments of CP- and GP-AFL techniques could be used to integrate nanochannels in complex microfluidic systems, such as lab-on-chip devices [40,41]. In addition, nanogrooves having continuously variable depth profiles could be suitable for generating single-photon emitter patterns with a nanometer-scale for the precise manipulation of two-dimensional materials [10,42].

## 4. Conclusions

In this work, a novel AFM nanolithography technique, based on nanoindentation and termed Pulse-AFL, was presented. With its two variants, Constant Pulse- and Gradient Pulse-AFL, we were able to fabricate in a soft material, i.e., PMMA, 2.5D nanogrooves with constant- and varying-depth, respectively. Nanostructures with high regular profiles were fabricated in a single-step process and without the support of other additional energy sources (thermal or vibrational), thereby, significantly reducing the manufacturing complexity. By tuning the machining parameters (i.e., the setpoint and its gradient) the nanogrooves features were controlled in a reproducible fashion.

Moreover, since the two types of lithography were different only in the choice of the pulse amplitudes, the switch from one to the other in the same process was smooth. Then, patterns with different depth (constant or varying) could be developed with continuity. Those well-defined structures were successively transferred onto a more resilient material (silicon nitride) by means of the ICP etching process with high fidelity, thus, preserving the shape and the bottom flatness of the nanochannels.

The developed lithographic technique will enable the fabrication of structures with different shapes, depth/height, and size by simply modifying some nanolithography inputs/parameters, such as the pattern template, pulses amplitude and frequency, and indentation step. Moreover, the demonstrated capability of transferring the shapes from the soft PMMA to a harder substrate adds considerable value in an industrial perspective.

## Figures and Tables

**Table 1:** Force values estimation by force spectroscopy.

Setpoint	1	2	3	4	5	6	7	8	9	10
Fz (µN)	2.14	3.89	5.68	7.45	9.12	10.91	12.62	14.33	16.06	17.94
St.Dv. (µN)	0.05	0.07	0.10	0.12	0.14	0.16	0.19	0.23	0.28	0.34
